# Characterization and Transcriptome Analysis of a Long-Chain *n*-Alkane-Degrading Strain *Acinetobacter pittii* SW-1

**DOI:** 10.3390/ijerph18126365

**Published:** 2021-06-11

**Authors:** Weina Kong, Cheng Zhao, Xingwang Gao, Liping Wang, Qianqian Tian, Yu Liu, Shuwen Xue, Zhuang Han, Fulin Chen, Shiwei Wang

**Affiliations:** 1 Key Laboratory of Resources Biology and Biotechnology in Western China, School of Life Sciences, Northwest University, Ministry of Education, Xi’an 710069, China; kongwn@nwu.edu.cn (W.K.); zhaocheng@stumail.nwu.edu.cn (C.Z.); wangliping@stumail.nwu.edu.cn (L.W.); tianqianqian@stumail.nwu.edu.cn (Q.T.); liuyu1@stumail.nwu.edu.cn (Y.L.); xuesw@nwu.edu.cn (S.X.); 2 Hulangmao Oil Production Area in No.3 Oil Production Plant of Changqing Oilfield Company, Yan’an 717500, China; gaoxw1632021@163.com; 3 Institute of Deep-Sea Science and Engineering, Chinese Academy of Sciences, Sanya 572000, China; hanz202105@163.com

**Keywords:** *Acinetobacter pittii*, long-chain *n*-alkanes, alkane hydroxylase, whole-genome sequencing, transcriptome analysis

## Abstract

Strain sw-1, isolated from 7619-m seawater of the Mariana Trench, was identified as *Acinetobacter pittii* by 16S rRNA gene and whole-genome sequencing. *A. pittii* sw-1 was able to efficiently utilize long-chain *n*-alkanes (C_18_–C_36_), but not short- and medium-chain *n*-alkanes (C_8_–C_16_). The degradation rate of C_20_ was 91.25%, followed by C_18_, C_22_, C_24_, C_32_, and C_36_ with the degradation rates of 89.30%, 84.03%, 80.29%, 30.29%, and 13.37%, respectively. To investigate the degradation mechanisms of *n*-alkanes for this strain, the genome and the transcriptome analyses were performed. Four key alkane hydroxylase genes (*alkB*, *almA*, *ladA1*, and *ladA2*) were identified in the genome. Transcriptomes of strain sw-1 grown in C_20_ or CH_3_COONa (NaAc) as the sole carbon source were compared. The transcriptional levels of *alkB* and *almA*, respectively, increased 78.28- and 3.51-fold in C_20_ compared with NaAc, while *ladA1* and *ladA2* did not show obvious change. The expression levels of other genes involved in the synthesis of unsaturated fatty acids, permeases, membrane proteins, and sulfur metabolism were also upregulated, and they might be involved in *n*-alkane uptake. Reverse transcription quantitative polymerase chain reaction (RT-qPCR) confirmed that *alkB* expression was significantly induced by C_20_, C_24_, and C_32_, and *almA* induction extent by C_24_ and C_32_ was higher than that with C_20._ Furthermore, *ladA2* expression was only induced by C_32_, and *ladA1* expression was not induced by any of *n*-alkanes. In addition, *A. pittii* sw-1 could grow with 0%–3% NaCl or 8 out of 10 kinds of the tested heavy metals and degrade *n*-alkanes at 15 °C. Taken together, these results provide comprehensive insights into the degradation of long-chain *n*-alkanes by *Acinetobacter* isolated from the deep ocean environment.

## 1. Introduction

The problem of offshore oil leakage during exploitation and transportation has considerably impacted the balance of marine natural ecology and the survival of human beings. Alkanes are the main component of petroleum pollutants. Several microorganisms in nature can degrade alkanes. To date, bacteria [1,2,3], fungi [4,5], and algae [6,7] have been reported to play roles in the degradation of alkanes. Microbes can transform alkanes into less harmful or non-hazardous substrates that can participate in natural biogeochemical cycles [8], and therefore, bioremediation is more economically and environmentally friendly compared with chemical and physical remediation methods. *Acinetobacter*, which is widely found in nature, is one of the dominant bacteria in soil and marine environments [9,10]. Recently, many reports have shown that *Acinetobacter* strains can degrade alkanes [11,12,13], indicating that *Acinetobacter* is promising for application in the repair engineering of alkane pollution, and more *Acinetobacter* strains with a high degradation efficiency of petroleum hydrocarbons are expected to be isolated.

Thus, it is of considerable significance to study the alkane degradation mechanisms of *Acinetobacter*. Diverse alkane hydroxylases have been characterized in several bacterial species. Cytochrome P450 mediates the oxidation of shorter chain alkanes (C_5_–C_18_) [14,15]. Alkane-1-monooxygenase (AlkB) has a wide range of alkane degradation activities. AlkB in *Pseudomonas putida* GPo1 has been characterized, and its activity helped utilize alkanes ranging from C_6_ to C_12_ [16]. *alkB1* and *alkB2* were obtained from *A. oleivorans* DR1. *alkB1* was responsible for long-chain (LC) alkane degradation (C_24_–C_26_), whereas *alkB2* participated in mid-chain alkane (C_12_–C_16_) utilization [17]. Additionally, the flavin-binding LC-alkane hydroxylase (AlmA) was first identified in *Acinetobacter* sp. strain DSM 17,874. It is responsible for LC alkane (C_32_ and longer) degradation [18]. Interestingly, the homolog of *almA* from *A. oleivorans* DR1 shows negligible activity for alkane degradation [17]. The thermophilic soluble LC-alkane hydroxylase (LadA) was first reported in *Geobacillus thermodenitrificans* NG80-2 and could degrade LC alkanes with carbon atom number at least up to 36 (C_36_) [19].

In this study, *A. pittii* sw-1 was isolated from 7619-m seawater in the Mariana Trench. The degradation potential was examined using different types of LC *n*-alkanes under various conditions. The genes involved in alkane degradation were analyzed via whole-genome sequencing, homologous alignment, and transcriptome analysis. Additionally, reverse transcription quantitative polymerase chain reaction (RT-qPCR) was carried out to verify the transcriptome analysis. These results provided insights into the LC *n-*alkane degradation by *Acinetobacter.*

## 2. Materials and Methods

### 2.1. Strain, Media, and Chemicals

Seawater samples from the Mariana Trench were collected using Tianya Lander during the TS01 voyage of the ocean research vessel Discovery No. 1 from 25 to 26 July 2016 (142°08.6521′ E, 11°04.7131′ N). Strain sw-1 was isolated from the 7619-m seawater sample using 2216E agar and it displayed round, smooth, and white colony. Luria–Bertani (LB) medium and basal salt medium (BSM) [20] were also used in this study. LB agar was used to resuscitate strains from the −80 °C refrigerator and BSM was used to provide basal salt in alkane degradation experiments of strain sw-1. All *n*-alkanes including C_8_–C_36_ were of analytical grade and obtained from Sigma-Aldrich Shanghai Trading (Shanghai, China). Various *n*-alkanes were first dissolved in *n*-hexane to prepare 100 g/L *n*-alkane solutions. Then, 250 μL of these solutions was added to 50 mL of BSM liquid to obtain 500 mg/L *n*-alkanes for degradation examination.

### 2.2. 16S rRNA Gene Analysis and Phylogenic Tree Construction

The 16S rRNA gene of strain sw-1 was PCR-amplified using the eubacterial primers 27F and 1492R (Table S1). Phylogenetic and molecular evolutionary of strain sw-1 and other strains was analyzed with Molecular Evolutionary Genetics Analysis (MEGA 6.0) using the neighbor joining method with 1000 bootstrap value and visualized using the Interactive Tree of Life (iTOL v5) [21]. The 16S rRNA gene sequence was deposited in GenBank (accession no. MT367764.1).

### 2.3. Genome Sequencing and Comparative Genomics Analysis

A whole-genome shotgun strategy was adopted to construct the genome library, and sequencing was carried out using next-generation sequencing (NGS) supported by Illumina NovaSeq 6000 technology. FastQC (version v0.11.5, Babraham Institute, Cambridge, UK) and Trimmomatic (version 0.38, Institute of Bio- and Geosciences: Plant Sciences, Julich, Germany) were used for performing read quality control and for trimming and removal of joint sequences, respectively. Thereafter, high-quality reads were obtained and assembled using shovill (version 1.0.4, The University of Melbourne, Melbourne, Australia) to generate the draft genome sequence of strain sw-1. Data from the whole-genome shotgun analysis were deposited in GenBank under the accession no. JABBFA000000000. Sequence similarity analysis was performed by comparing homologous fragments between the genomes using Orthologous Average Nucleotide Identity (OrthoANI, Department of ChunLab, Seoul National University, Seoul, Republic of Korea) [22]. Based on 16S rRNA gene analysis result, 9 representative *Acinetobacter* strains with the most similarity or relativity with strain sw-1 were selected, and the data on the genomes of the 9 *Acinetobacter* strains were obtained from NCBI.

### 2.4. Growth and Biodegradation Rate of SW-1 in the Presence of Various n-Alkanes

The biodegradation of *n*-alkanes was performed in a liquid culture using washed cell suspensions. Briefly, a single colony of strain sw-1 was cultivated in 5 mL of LB broth overnight. The cells were then harvested and subjected to washing three times with PBS buffer and resuspended in PBS buffer to obtain an OD_600_ of approximately 1.0. Subsequently, 500 μL of the cell inoculum was inoculated into 50 mL of BSM containing 500 mg/L of various *n*-alkanes (C_8_–C_36_) [20]. Flasks containing cells but no *n*-alkanes and those containing *n*-alkanes, but no cells were both used as control groups. All flasks were incubated at 30 °C for 7 d. The growth of the strain was determined by measuring the OD_600_ of the cell cultures [23]. After cultivation, the flasks containing residual alkanes were placed in the 4 °C refrigerator, which made alkanes become solid. Ten-milliliter *n*-hexane was added to the flasks, followed by being shaken vigorously for 10 min and set for 10 min. Then, the mixed liquid was centrifuged for 10 min at 10,000 rpm and organic phases were collected as extracts. In addition, the flasks and tubes were washed with *n*-hexane and integrated into extracts. All extracts were collected and placed in a fume cupboard to vaporize the solvent with nitrogen to obtain solid alkanes and then dissolved in 5 mL of n-hexane [24]. The obtained *n*-hexane was passed through a 0.22-μm organic membrane filter and taken as detected samples for gas chromatograph analysis. Flasks containing *n*-alkanes without bacterial cells were treated with the same procedure and the corresponding samples were taken as initial mass. The samples were detected using a 7890A gas chromatograph (Agilent, Santa Clara, CA, USA) with FID using a capillary BP5 column (30 m × 0.32 mm × 0.25 μm). The injector temperature and detector temperature were 250 and 310 °C, and the oven temperature was kept at 80 °C for 5 min and then raised to 300 °C at a rate of 10 °C/min [25]. The degradation rate of alkanes was determined by deducting the mass of residual alkanes from the initial mass. All experiments were repeated three times, and the results have been represented as mean values.

### 2.5. RNA-Seq and Data Analysis

Strain sw-1 was grown in three independent samples using two different carbon sources, namely C_20_ (500 mg/L) and CH_3_COONa (NaAc) (2 g/L), and the cultured cells were collected at mid-log phase. Thereafter, the total RNA was extracted using the Trizol reagent (Invitrogen, Carlsbad, CA, USA), and DNase I was used to eliminate genomic DNA contamination. The RNA-seq strand-specific libraries were prepared according to the instructions of the RNA sample preparation kit (San Diego, CA, USA) using 5 μg of total RNA. Transcriptional abundance was used as an input to DESeq (version 1.18.0, University of Duisburg-Essen, Essen, Germany) to assess differential expression. Transcripts with |log_2_ fold change| > 1 and *p* < 0.05, were selected as remarkably induced genes. A volcano map was illustrated using the R language.

### 2.6. RT-qPCR

Strain sw-1 was grown in BSM with NaAc and *n*-alkanes, respectively. Approximately 10^8^ cells were harvested and collected. Total RNA was extracted using the Total RNA Micro Kit (Magen, Guangzhou, China). Then, 1 μg of RNA was used to synthesize cDNA using the Prime Script TMRT Reagent Kit (TaKaRa, Dalian, China). Primers used in this study are listed in Table S1. RT-qPCR was performed using the BioRad CFX96 Real-Time PCR Detection System (Hercules, CA, USA). The reactions were conducted in a 10-μL reaction volume including the DNA template, 5 μL SYBR Green PCR Super mix, primers, and sterile water. RT-qPCR was performed under the following conditions: 5 min at 95 °C, 15 s at 95 °C, 30 s at 57 °C, and 30 s at 72 °C, with a total of 45 cycles. The expression levels of the investigated genes were normalized to those of *rpoB* gene [26]. The relative expression levels of target genes were determined using the 2^−^^△△Ct^ method [27].

### 2.7. Effect of Rhamnolipid, NaCl, pH, and Temperature on C_20_ Utilization by Strain SW-1

Fermentation was conducted by adding rhamnolipids at final concentrations of 50, 100, 200, and 400 mg/L to study the effect on C_20_ degradation by strain sw-1. To investigate the tolerance of bacteria to low temperature, pH, and NaCl, different temperatures (15 °C and 30 °C), pH values (4, 5, 6, 7, 8, 9, and 10), and concentrations of NaCl (0%, 1%, 2%, 3%, 4%, and 5%) were considered to detect the effects on bacterial growth. Other culture conditions were the same as described in Section 2.4.

### 2.8. Heavy Metal Tolerance Assays

The tolerance of strain sw-1 was detected using ten types of heavy metal salts (Fe^2+^, Mn^2+^, Cr^2+^, Co^2+^, Ni^2+^, Cu^2+^, Pb^2+^, Sr^2+^, Al^2+^, and Zn^2+^) as previously reported [28]. The concentrations of the metal salts were 100 μM. Strain sw-1 was inoculated into BSM with 500 mg/L C_20_ containing the above-mentioned individual metal salts. Strain sw-1 grown with 500 mg/L C_20_ was used as a control group. The culture conditions were the same as described in Section 2.4.

### 2.9. Determination of the Surface Tension and Cell Surface Hydrophobicity

The surface tension of the fermentation broth and cell surface hydrophobicity was measured to study the uptake mechanism of alkanes in strain sw-1. The surface tension was measured using a tensiometer (JYW-200A Automatic Interface Tensiometer, Xiamen, China) according to the previous report [29]. Briefly, the fermentation broth was transferred to a 50 mL tube, centrifuged at 8000 rpm for 5 min, and the supernatant was obtained. A glass beaker (25 mL) containing 20 mL of cell-free supernatant was placed onto the tensiometer platform. A platinum plate was slowly touched to the liquid–air interface to measure the surface tension. The instrument was calibrated by measuring the surface tension of pure water prior to each experiment. The experiment was repeated three times. Cell surface hydrophobicity was determined by assessing microbial adherence to *n*-octane [30]. Briefly, the bacterial cells were subjected to washing twice with PUM buffer and suspended at an OD_600_ of 0.6; then, 1 mL of the suspension was mixed with 0.2 mL of *n*-octane. The water phase at the bottom was considered for the measurement of OD_550_ after allowing the mixture to stand for 1 h. The hydrophobicity was determined using the formula (1 − A/A_0_) × 100%. A represents the OD_550_ of the water phase after treatment, and A_0_ represents the OD_550_ of the bacterial suspension before treatment.

## 3. Results

### 3.1. Isolation and Identification of A. pittii Strain SW-1

**Figure 1:**
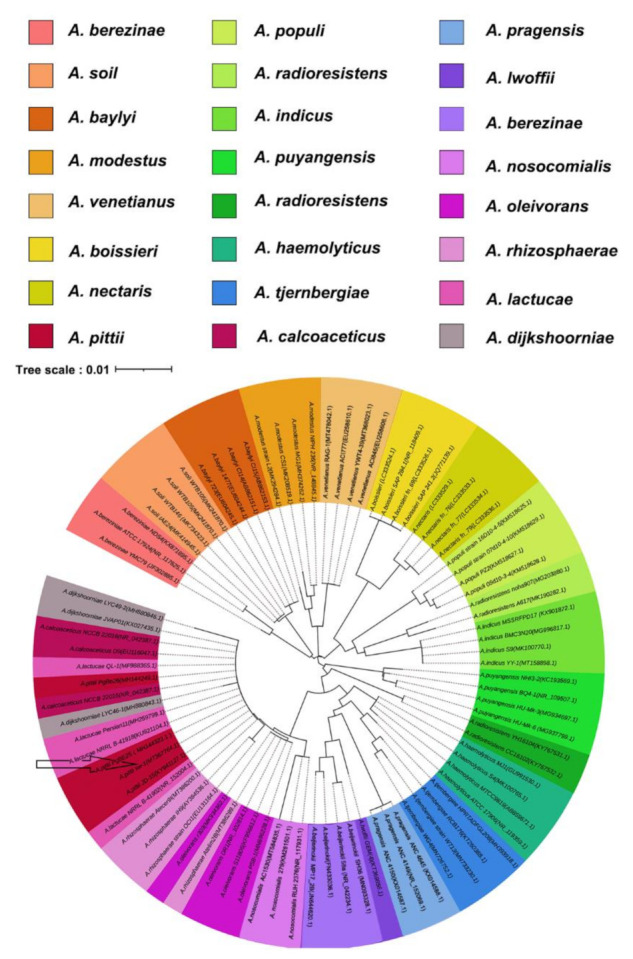
Phylogenetic tree analysis of *Acinetobacter* strains based on 16S rRNA gene sequencing. The gene sequence of strain sw-1 was compared with that of 23 *Acinetobacter* isolates to determine its evolutionary position. Data for all 16S rRNA genes were downloaded from NCBI. Phylogenetic and molecular evolutionary analysis was conducted at 1000 bootstrap value using the MEGA software and was visualized by using iTOL.

The strain used in this study was isolated from 7619-m seawater in the Mariana Trench and named sw-1. The 16S rRNA gene sequence of sw-1 showed 100% identity with that of *A. pittii* PgBE252, and phylogenetic tree analysis showed a close relationship between sw-1 and other *Acinetobacter* strains (Figure 1). Orthologous Average Nucleotide Identity (OrthoANI) values were used to identify evolutionary relationships among strains by performing paired comparison of genes existing in all the strains. To determine the evolutionary relationship of strains at the genome level, nine *Acinetobacter* strains were selected for comparison. The similarity values between these strains ranged from 74.82%−99.84% (Figure S1). Species with the values above 95% can be considered as the same species [31], and therefore, sw-1 was classified as an *A. pittii* strain.

### 3.2. A. pittii SW-1 Degrades LC n-Alkanes

**Figure 2:**
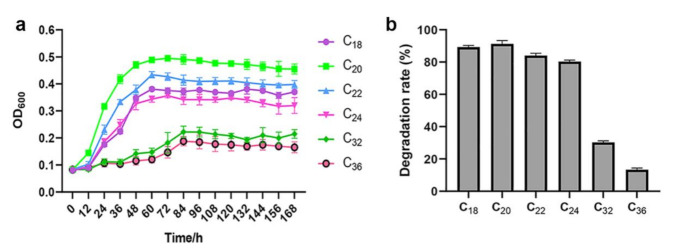
(**a**) Growth curves of sw-1 in 500 mg/L of various *n*-alkanes (C_18_–C_36_). Strain sw-1 was inoculated in 50 mL of BSM containing 500 mg/L of *n*-alkanes. All flasks were incubated at 30 °C for 7 d. The strain growth was determined by estimating OD_600_ of cells every 12 h. (**b**) Degradation rate of various *n*-alkane. The residual alkanes in BSM were extracted using *n*-hexane and were detected using gas chromatograph, and the degradation rate of alkanes was determined by deducting the mass of residual alkanes from the initial mass.

The degradation of *n*-alkanes by *A. pittii* sw-1 was assessed by detecting the growth of the strain within 7 d in BSM containing 500 mg/L of various *n*-alkanes (C_8_–C_36_). Among them, strain sw-1 showed the best growth under C_20_, followed by C_22_, C_18_, C_24_, C_32_, and C_36_ (Figure 2a). Strain sw-1 showed no growth in the presence of shorter *n*-alkanes (C_8_–C_16_) as the only carbon source. The alkanes degradation rate on the seventh day of C_18_–C_24_ was above 80%, and the degradation efficiency of C_20_ was as high as 91.25%, while the degradation rates of C_32_ and C_36_ were only 30.29% and 13.37%, respectively (Figure 2b).

### 3.3. Analysis of the Genes Involved in Alkane Degradation

**Figure 3:**
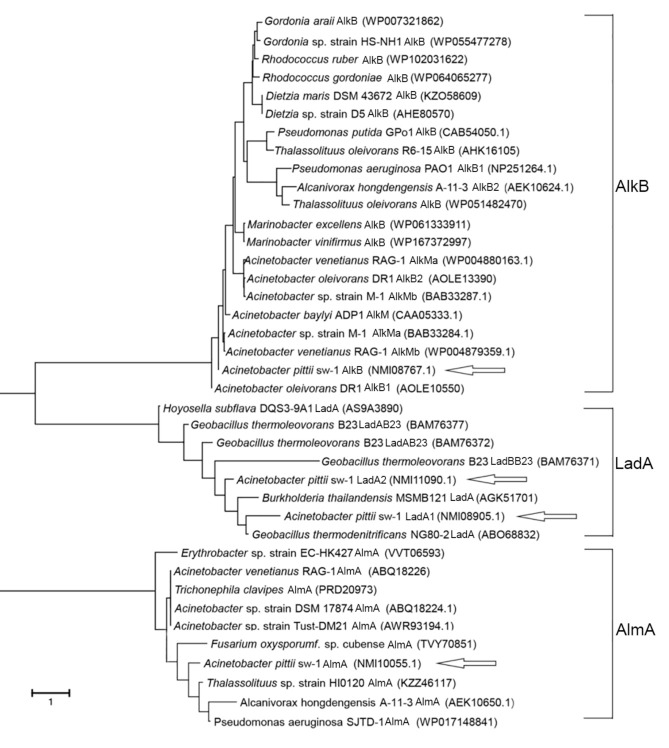
Phylogenetic analyses of AlkB, AlmA, LadA1, and LadA2 in *A. pittii* sw-1 compared with homologous proteins in other bacteria. Phylogenetic and molecular evolutionary analysis was conducted at a 1000 bootstrap value using the MEGA software. Data on the sequences of other genes were downloaded from NCBI.

To identify the alkane hydroxylase involved in alkane metabolism in *A. pittii* sw-1, after genome sequencing, sequence analysis was performed through homologous amino acid sequence alignment using CLUSTALW (available online: https://www.genome.jp/tools-bin/clustalw (accessed on 1 November 2019)). Four alkane hydroxylases, AlmA, AlkB, LadA1, and LadA2, that probably participated in the utilization of LC alkanes, were identified in *A. pittii* sw-1. We constructed phylogenetic trees of four alkane hydroxylases compared with homologous proteins in other bacteria (Figure 3). The protein AlkB displayed 95.58% and 61.04% amino acid identity with AlkB1 and AlkB2 in *A. oleivorans* DR1 (Table 1). AlmA showed 81.49% amino acid identity with the homologous gene in *Acinetobacter* sp. strain DSM 17874 (Table 1). LadA1 and LadA2 in *A. pittii* sw-1 displayed 45.44% and 49.78% amino acid identity with LadA from *G. thermodenitrificans* NG80-2, respectively (Table 1). It has been demonstrated that RubA and RubB are the main components of the electron transport chain for alkane degradation by *Acinetobacter* strain ADP1 [32]. Two homologous proteins, RubA and RubB, were also found in *A. pittii* sw-1, which showed 94.44% and 82.95% amino acid identity, respectively, with those in *Acinetobacter* sp. strain ADP1. An alkane utilization regulator, AlkR, was also found in the genome of *A. pittii* sw-1, which belongs to the AraC family transcriptional regulator.

### 3.4. Alkane Degradation-Related Genes Were Induced by C_20_

**Figure 4:**
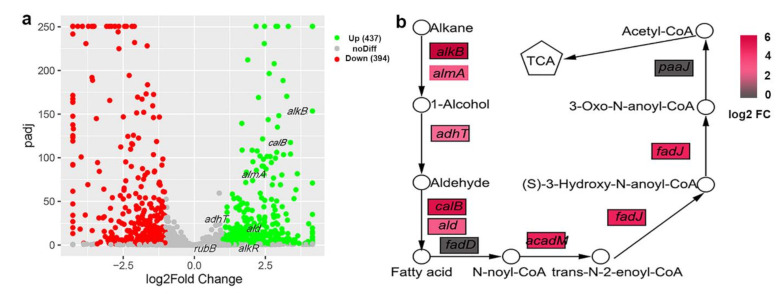
(**a**) The volcano plot illustrating the expression levels of the genes with significant difference in alkane degradation. Several genes involved in alkane metabolism were labeled. *alkB*, alkane hydroxylase gene; *alma*, flavin-binding monooxygenase gene; *adhT*, alcohol dehydrogenase gene; *ald*, aldehyde dehydrogenase gene; *alkR*, alkane hydroxylase transcriptional regulator gene; *rub*, rubredoxin reductase gene. (**b**) Pathway of alkane metabolism and related genes involved in alkane oxidation and β-oxidation. *calB*, aldehyde dehydrogenase gene; *fadD*, acyl-CoA synthetase gene; *acadM*, enoyl-CoA hydratase gene; *fadJ*, 3-hydroxybutyryl-CoA epimerase gene; *paaJ*, beta-ketoadipyl CoA thiolase gene. Upregulated genes are marked in red, and the non-induced genes are indicated in gray. A deeper color used in the illustration represents stronger induction by C_20_.

RNA-Seq was performed using C_20_ or NaAc as the sole carbon source. A total of 831 genes were found to be differentially expressed. Among them, the expression levels of 437 genes were upregulated (Figure 4a). The results showed that the expression of numerous alkane degradation-related genes was altered in the presence of C_20_. the expression levels of alkane hydroxylase genes, *alkB* and *almA*, were upregulated 78.28-fold and 3.51-fold in C_20_, respectively, while those of *ladA1* and *ladA2* were not significantly increased (Figure 4a) (Table S2). Higher *alkB* expression indicated that AlkB could be the main alkane hydroxylase involved in C_20_ degradation. Additionally, the expression of the alkane utilization regulator gene *alkR* was 2.66-fold upregulated, while the expression of *rubA* and *rubB* was not induced (Figure 4a) (Table S2). Furthermore, the expression of one alcohol dehydrogenase gene, *dhaT*, and two aldehyde dehydrogenase genes, *calB* and *ald,* was induced in the presence of C_20_ (Figure 4b) (Table S2). The fatty acids produced by alkane metabolism might act as energy sources for bacteria through β-oxidation. Indeed, the expression levels of the acyl-CoA dehydrogenase gene *acadM* and enoyl-CoA hydratase gene *fadJ* were also upregulated (Figure 4b) (Table S2).

Under conditions of C_20_-mediated induction, the expression levels of fatty acid desaturase and delta-9 acyl-lipid fatty acid desaturase, which participate in the synthesis of unsaturated fatty acids, were upregulated 17.95-fold and 3.34-fold, respectively (Table S2). The expression levels of genes *des*6 and *hmp*, responsible for catalyzing dehydrogenation from C_9_, were also significantly upregulated, and the expression levels of *tesB* and *yciA* converting palmitoyl-CoA into palmitic acid were downregulated (Table S2).

Transcriptome analysis also showed that the expression levels of 12 genes encoding membrane proteins and four genes encoding permeases were upregulated (Table S2). The expression levels of two sulfate transport-related genes, *cysT* and *cysP*, were upregulated 74.13-fold and 5.03-fold, respectively. The expression of the thioredoxin reductase gene was upregulated 22.93-fold, and the expression of sulfate adenylyl transferase gene *cysD* was upregulated 8.62-fold (Table S2).

### 3.5. Detection of the Expression of Alkane Degradation-Related Genes by RT-qPCR

**Figure 5:**
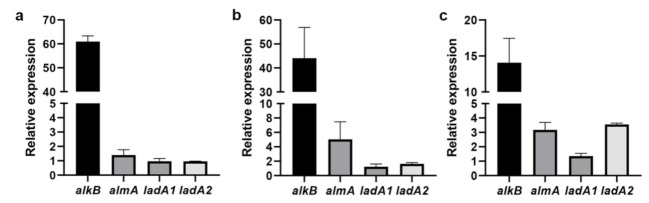
The relative expression levels of four alkane hydroxylase genes in C_20_ (**a**), C_24_ (**b**), and C_32_ (**c**) detected by RT-qPCR analysis. The relative expression levels of four alkane hydroxylase genes in diverse alkanes (C_20_, C_24_, C_32_) to the expression in AcNa were normalized by that of *rpoB*. The 2^−^^△△Ct^ method was used to calculate the fold change of the normalized mRNA expression levels.

RNA-Seq analysis revealed that the expression of several alkane degradation-related genes was induced by C_20_. To confirm the results of the transcriptome analysis, total RNA was extracted from sw-1 cells induced by different *n*-alkanes (C_20_, C_24_, and C_32_) and NaAc, and the expression levels of *alkB*, *almA*, *ladA1*, and *ladA2* were determined by performing RT-qPCR. These results showed that *alkB* was remarkably induced by C_20_, C_24_, and C_32_ (Figure 5). *almA* was induced 5.03-fold and 3.17-fold in the presence of C_24_ and C_32_, respectively, and 1.40-fold in the presence of C_20_. *ladA2* expression was upregulated 3.56-fold in the presence of C_32_, while it did not show remarkable change in the presence of C_20_ and C_24_ (Figure 5). *ladA1* expression was not induced in the presence of any alkane (Figure 5). These results also suggested that alkane hydroxylase AlkB could play a predominant role in alkane degradation for *A. pittii* sw-1.

### 3.6. Effects of Rhamnolipids, NaCl, pH, Low Temperature, and Heavy Metals on A. pittii SW-1 Growth

**Figure 6:**
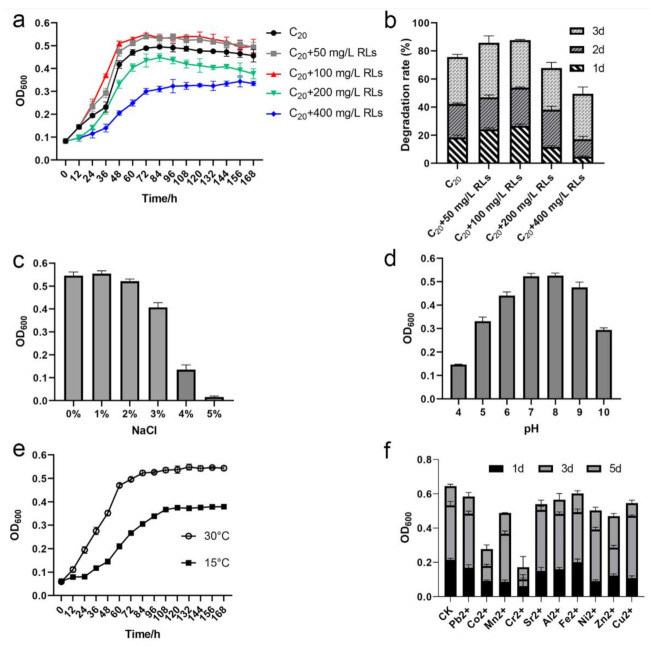
The growth curves of *A. pittii* sw-1 under various conditions and its alkane degradation rate. (**a**) Growth of *A. pittii* sw-1 in 500 mg/L C_20_ with 0–400 mg/L rhamnolipids (RLs). Strain sw-1 was inoculated in 50 mL of BSM with 500 mg/L C_20_. All flasks were kept at 30 °C for 7 d. The growth was determined by examining OD_600_ every 12 h. The daily degradation rate of C_20_ for 3 d was measured after adding different concentrations of RLs, and then compared with the degradation rate of the control group without RLs. (**b**) Degradation rate of C_20_ with different concentrations of RLs. (**c**) Growth of *A. pittii* sw-1 in C_20_ with 0–5% NaCl. (**d**) Growth of *A. pittii* sw-1 in 500 mg/L C20 with various pH values (4–10) for 7 d. (**e**) Growth of *A. pittii* sw-1 in 500 mg/L C_20_ with two kinds of temperature (30 °C and 15 °C) for 7 d. (**f**). Growth of *A. pittii* sw-1 in 500 mg/L C_20_ with diverse heavy metals. The concentration of each heavy metal was 100 μM. Bacterial growth on 1st, 3rd, and 5th d was compared. All experiments were repeated three times. The error bars represent standard errors.

Surfactants increase the solubility of hydrophobic substances in water, enabling microorganisms to metabolize these substances more efficiently [36]. Therefore, we examined the effects of rhamnolipids on the growth and degradation rates of *A. pittii* sw-1 with C_20_. When rhamnolipids were added at concentrations ranging from 50–100 mg/L, the growth of *A. pittii* sw-1 was promoted, while a concentration more than 200 mg/L inhibited its growth (Figure 6a). The degradation rate was also enhanced by adding 50–100 mg/L rhamnolipids, while the addition of 200–400 mg/L rhamnolipids inhibited the degradation of C_20_ (Figure 6b). The degradation efficiency increased from 18.47%–26.66% with 100 mg/L rhamnolipids on the first day, and from 75.57%–87.65% on the third day (Figure 6b).

*A. pittii* sw-1 was isolated from a deep ocean environment; hence, the influence of NaCl, pH, and low temperature on *A. pittii* sw-1 was assessed. *A. pittii* sw-1 showed obvious growth under the conditions of 0%–3% NaCl. When the concentration of NaCl was higher than 3%, the growth of strain sw-1 was inhibited, and 5% NaCl completely inhibited its growth (Figure 6c). The optimum pH range for its growth was 7~8 (Figure 6d). In addition, although the growth of *A. pittii* sw-1 appeared weaker at 15 °C than that at 30 °C, it still exhibited obvious growth and alkane degradation ability (Figure 6e). In order to examine the effects of heavy metals on *A. pittii* sw-1 degradation ability, the tolerance of *A. pittii* sw-1 to heavy metals was also evaluated. The results showed that Co^2+^ and Cr^2+^ obviously inhibited the growth of *A. pittii* sw-1, while the other heavy metals tested in the study had little effect on its growth (Figure 6f). Furthermore, we also identified several genes that might be involved in salt tolerance (Table S3) and cold adaption in *A. pittii* sw-1 (Table S4), consistent with the above-mentioned results. In general, *A. pittii* sw-1 exhibited tolerance to low temperatures (15 °C), eight of ten tested heavy metals, and 1%–3% NaCl.

### 3.7. Cell Surface Hydrophobicity Might Contribute to the Alkane Uptake in A. pittii SW-1

**Figure 7:**
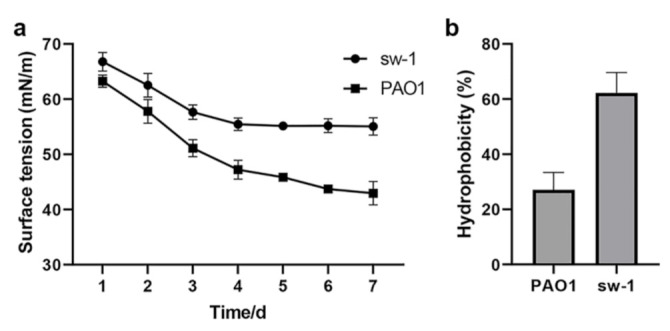
The difference between *A. pittii* sw-1 and *P. aeruginosa* PAO1 in terms of fermented liquid surface tension (**a**) and cell surface hydrophobicity (**b**). The determination of surface tension was conducted using a tensiometer. The measurement was performed once a day. The cell surface hydrophobicity was detected by assessing cell adherence to *n*-octane.

Alkanes are a class of hydrophobic organic compounds that cannot be easily dissolved in water [37]. Biological surfactants can be used to reduce the surface tension of liquids and emulsify alkanes, which facilitate alkane accessibility for bacteria. Bacteria also can utilize alkanes by absorbing them directly through cell surface hydrophobicity [38]. Therefore, low surface tension and high cell surface hydrophobicity are favorable for absorbing alkane. To investigate the uptake mechanism of alkanes in *A. pittii* sw-1, the surface tension of the fermentation broth and cell surface hydrophobicity were comparatively analyzed with the common alkane degradation strain *Pseudomonas aeruginosa* PAO1. The results showed that the liquid surface tension and the cell surface hydrophobicity of strain sw-1 were both higher compared with those of *P. aeruginosa* PAO1, indicating strain sw-1 might absorb alkane through cell surface hydrophobicity (Figure 7).

## 4. Discussion

*Acinetobacter* is an important genus that exhibits the excellent ability of alkane biodegradation in the environment. Several strains of this genus have been reported to be alkane utilizers. For example, previous studies showed that *A. calcoaceticus* S30 was able to degrade C_17_–C_24_ [39] and *A. oleivorans* DR1 degraded C_12_–C_30_ [17]. Strain sw-1 was isolated from seawater at a depth of 7619-m in the Mariana Trench and belonged to *A. pittii.* It could utilize LC *n*-alkanes (C_18_–C_36_) but not shorter chain *n*-alkanes (C_8_–C_16_). This might be attributable to the lack of the enzymes that are responsible for degrading short-chain alkanes, such as cytochrome P450. This was consistent with the genome analysis results. In addition, strain sw-1 displayed the highest utilization efficiency of C_20_ among the tested *n*-alkanes. It was different from the other reported *A. pittii* strains. For example, *A. pittii* strain ABC, isolated from oil sludge pits at Noonmati Refinery of India, showed the best growth in C_16_ [28], whereas strain sw-1 could not utilize C_16_ as the sole carbon source. The different alkane degradation characterization of these two strains might be due to different isolation environments and substrate bias of alkane hydroxylases.

Several alkane hydroxylases have been identified in *Acinetobacter* and other bacteria (Table 1). Among them, AlkB of strain sw-1 showed the most similarities to AlkB1 of *Acinetobacter* DR1 and AlkMa of *Acinetobacter* M-1 (Figure 3). Our data showed that the alkane degradation ability of AlkB in strain sw-1 was also similar with that observed in *Acinetobacter* DR1 and *Acinetobacter* M-1, and their expression was significantly induced by C_20_–C_32_ (Table 1) [17,33]. The LC alkane hydroxylase, AlmA, was first identified in *Acinetobacter* DSM 17,874 and could degrade *n*-alkanes with a carbon atom number more than 32 (>C_32_) [18]. The degradation profiles of AlmA display some small differences for various species. In *Alcanivorax Hongdengensis* A-11-3 AlmA was shown to be involved in C_18_–C_36_ degradation, especially with higher induced expression for C_24_–C_36_ [35], AlmA of *A. dieselolei* B-5 was only induced by C_22_–C_36_, especially with higher expression in the degradation of C_28_–C_36_ [40], in *P. aeruginosa* SJTD-1 AlmA was be induced by C_18_–C_24_ [20], and in *P. aeruginosa* DN1 AlmA was induced by C_20_ and longer chain *n*-alkanes but not shorter chain n-alkanes [23]. Phylogenetic tree analysis suggested that AlmA in strain sw-1 was close to that in *Acinetobacter* strains, far from those in *Alcanivorax* and *Pseudomonas* spp., and AlmA expression in strain sw-1 was induced by longer chain alkanes (C_24_ and C_32_). These results indicated that AlmA in diverse strains all played roles in the degradation of LC alkanes, although some small differences were found. Another LC alkane hydroxylase LadA, firstly isolated from in *G. thermodenitrificans* NG80-2, was able to oxidize C_15_–C_36_ alkanes [19]. Strain sw-1 has two LadA, LadA1 and LadA2. LadA2 expression was induced only by C_32_, while *ladA1* expression was not obviously induced by any tested alkanes (Figure 5). In *G. thermodenitrificans* B23, three LadA-type alkane monooxygenases were found to utilize various alkanes from C_12_ to C_23_ [41]. LadA are also identified from other bacteria such as *Octadecabacter antarcticus* and *Terroglobus saanensis* [42], while the function of these proteins is yet to be examined.

Biosurfactants are environmentally friendly and can be easily degraded, which facilitates their application in the bioremediation of petroleum hydrocarbon pollution [43]. Rhamnolipids, as glycolipid biosurfactants, can be used to increase the solubility of hydrocarbons in the aqueous phase by reducing the interfacial tension [44], and have been shown to promote the dissolution of hydrocarbons and to improve their bioavailability [45,46]. Our results showed that a low concentration (50–100 mg/L) of rhamnolipids promoted the biodegradation of alkanes in strain sw-1, while higher concentrations of rhamnolipids inhibited the growth of sw-1 and subsequent alkane biodegradation (Figure 6a,b). This indicated that a high concentration of rhamnolipids might be toxic to strain sw-1, consistent with the antimicrobial activity reported in other bacteria [47].

The expression of unsaturated fatty acid genes and synthesis of unsaturated fatty acids were also induced by C_20_ (Table S2). These results suggested that the strain might enhance cell membrane fluidity by facilitating the synthesis of such fatty acids to absorb alkanes. When strain sw-1 utilizes C_20_, it might increase the cell demand for sulfur. Transcriptome analysis of *A. oleivorans* DR1 showed that the expression levels of sulfate transport genes *cysAWUP* were upregulated by C_16_ [48]. We obtained similar results for strain sw-1. Levels of two sulfate transport proteins, *cysT* and *cysP*, and a sulfate adenylyl transferase *cysD*, were upregulated by C_20_ in strain sw-1 (Table S2). Bacteria absorb alkanes through their cell membranes, and membrane permeability is of substantial significance for the utilization of alkanes [49]. Thus, permeases and membrane proteins play an important role in the process of alkane absorption, transport, and regulation of the cell’s corresponding external environment. Transcriptome analysis also showed that certain genes encoding membrane protein permeases were induced by C_20_ (Table S2).

Lastly, it is worth mentioning that strain sw-1 was isolated from 7619-m seawater of the Mariana Trench, an extreme environment with a low temperature, high salinities and high hydrostatic pressures [50]. In general, microorganisms cannot survive in the environment except some extreme microorganisms. When cultured in the common laboratory with atmospheric pressure strain, sw-1 showed obvious growth, indicating that some gene mutation might have occurred to adapt to the standard laboratory culture conditions. This phenomenon was frequently found when the microbes from deep-sea were cultured in the standard laboratory [51,52]. In addition, alkanes are less found in deep-sea environments, but serpentinization and biological processes may release alkanes to the deep waters especially in subduction zones [53]. Heavy oil fractions such as LC alkanes may attach to suspended sediments and be deposited to the seabed and deep-sea water [54]. Oil contamination enhances the exo-polysaccharide production of phytoplankton [55], and its amphiphilicity promoted the dissolution of oil in sea water [56,57]. These results may be the reasons that many alkane-degrading bacteria have been isolated from deep-sea environments [58,59,60]. Furthermore, some excellent characteristics were maintained by strain sw-1, and it was able to grow under conditions of 1%–3% NaCl and utilize alkanes at a low temperature of 15 °C (Figure 6c,e), indicating strain sw-1 is promising for application in high salt and low temperature environments.

## 5. Conclusions

In conclusion, we isolated a LC alkane-degrading bacterium, strain sw-1, from the Mariana Trench, which was identified as *A. pittii.* Four alkane hydroxylases were identified via whole-genome sequencing and homologous alignment. The transcriptome analysis and RT-qPCR results showed that AlkB performed a major function in the utilization of alkanes, and AlmA and LadA2 were probably involved in the utilization of longer chains alkanes. Furthermore, strain sw-1 exhibited tolerance to low temperatures, heavy metals, and higher salt, and might be utilized in oil pollution remediation processes in similar environments.

## Figures and Tables

**Table 1:** The homologous alignment of functional alkane hydroxylases between *A. pittii* sw-1 and other bacteria using BLASTP.

Strain Name	Gene Name	Responsible for Range of Alkanes	Amino Acid Identity (%)	References
*Acinetobacter* M-1	*alkMa*	C_22_–C_30_	86.27 %	[33]
*Acinetobacter* M-1	*alkMb*	C_16_–C_22_	60.79 %	[33]
*Acinetobacter* DR1	*alkB1*	C_24_–C_26_	95.58 %	[17]
*Acinetobacter* DR1	*alkB2*	C_12_–C_16_	61.04 %	[17]
*Acinetobacter* ADP1	*alkM*	C_12_–C_18_	83.09 %	[11]
*Acinetobacter* RAG-1	*alkMa*	C_12_	61.04 %	[34]
*Acinetobacter* RAG-1	*alkMb*	C_12_	86.42 %	[34]
*Acinetobacter* DSM 17,874	*almA*	C_32_ and longer	81.49 %	[18]
*Alcanivorax Hongdengensis* A-11-3	*almA*	C_18_–C_36_	50.20 %	[35]
*P. aeruginosa* SJTD-1	*almA*	C_18_–C_24_	48.50 %	[20]
*G. thermodenitrificans* NG80-2	*ladA*	C_15_–C_36_	45.44% (with *ladA1*) 49.78% (with *ladA2*)	[19]

## Data Availability

The data presented in this study are available upon reasonable request from the corresponding author.

## Supplementary Materials

The following are available online at https://www.mdpi.com/article/10.3390/ijerph18126365/s1, Figure S1: the phylogenetic tree analysis based on the whole genome sequences of *Acinetobacter* strains; Table S1: the 16s RNA and RT-qPCR primers for alkane hydroxylase gene expression detection; Table S2: the alkane-degrading-related genes that could be induced by C_20_; Table S3: the salt tolerance-related genes in *A. pittii* sw-1; Table S4: the cold tolerance-related genes in *A. pittii* sw-1.
