# Supplementary material for: Characterization and Transcriptome Analysis of a Long-Chain n-Alkane-Degrading Strain Acinetobacter pittii SW-1
Source: Int J Environ Res Public Health. 2021 Jun 11;18(12):6365. doi: 10.3390/ijerph18126365 (PMC8296198; doi:10.3390/ijerph18126365)
Supplement: Supplementary file 1 [file ijerph-18-06365-s001.zip › ijerph-1206756-supplementary.pdf]

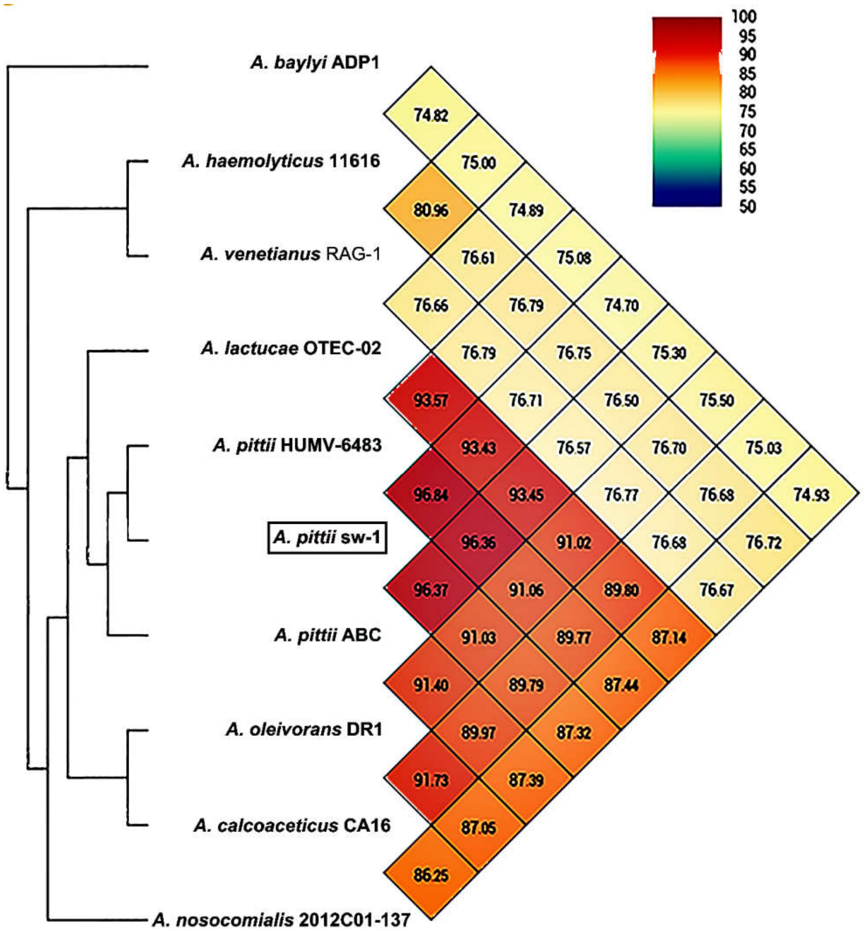

Figure S1. The phylogenetic tree analysis based on the whole genome sequences of *Acinetobacter* strains. The strain sw-1 labeled with a red background was compared with 9 strains of *Acinetobacter*. The other sequenced genomes were download from NCBI. Heatmap generated by comparison of genes with OrthoANI values were calculated by the OAT software. Different colors represent the distance of evolutionary relationships, while blue color represents distant relationships and red color represents close relationships.

Table S1. The 16s RNA and RT-qPCR primers for alkane hydroxylase gene expression detection.

Gene	Primers	Sequence (5′–3′)
16s RNA	27F	AGAGTTTGATCCTGGCTCAG
	1492R	TACGGYTACCTTGTTACGACTT
rpoB	*rpoB*-S	CCTACTCTACGTGCGGATAAAC
	rpoB-AS	CGTATTCAATTGCACCACCAC
alkB	*alkB*-S	GTACGTGGCCAGAACTCATAAA

	alkB-AS	CCATTTCCGTATCGAACATCCT
almA	*almA*-S	CCCATCTGGCACTACACATAAA
	almA-AS	CGCTGTCTCAGAAACATCCTAA
*ladA*1	*ladA1*-S	CAAGGTCGTATTGGCTGGAATA
	ladA1-AS	AACTCTTCGGCTTGCTCATAG
*ladA*2	*ladA2*-S	CCGACCAGCCTGACAATAAT
	ladA2-AS	CGCGATCCGTACTCAGTTAAA

Table S2. The alkane degrading related genes that could be induced by C_{20}.

Locus_tag	Gene name	Gene product	Fold Change
HHL03_04355	*alkB*	terminal alkane-1-monooxygenase	78.28
HHL03_11035	*almA*	putative flavin-binding monooxygenase	3.51
HHL03_04360	*alkR*	DNA-binding transcriptional regulator AraC	2.66
HHL03_07220	*dhaT*	putative alcohol dehydrogenase	2.13
HHL03_03455	*calB*	coniferyl aldehyde dehydrogenase (CALDH)	38.45
HHL03_14160	*ald*	aldehyde dehydrogenase	3.48
HHL03_07600	*fat*	fatty acid desaturase	17.95
HHL03_04350	*acadM*	acyl-CoA dehydrogenase	6.06
HHL03_12070	*fadJ*	enoyl-CoA hydratase	6.92
HHL03_00605	*desC*	delta-9 acyl-lipid fatty acid desaturase	3.34
HHL03_01775	*des6*	linoleoyl-CoA desaturase	5.57
HHL03_01780	*hmp*	flavodoxin reductase	3.64
HHL03_12425	*yciA*	acyl-CoA thioesterase	-3.89
HHL03_11225	*tesB*	acyl-CoA thioesterase II	-4.08
HHL03_12690	*des6*	linoleoyl-CoA desaturase	7.16
HHL03_02485	*hmp*	flavodoxin reductase	4.00
HHL03_19130	*tolC*	putative outer membrane secretion protein	10.57
HHL03_16210	*yidC*	inner membrane protein translocase component	3.45
HHL03_06105		membrane protein	3.47
HHL03_09275	*bamA*	putative outer membrane protein	2.25
HHL03_01730	*pstC*	high-affinity phosphate transport protein (ABC superfamily, membrane)	2.03
HHL03_02380	*pagO*	integral membrane protein	2.52
HHL03_08785	*yccS*	TIGR01666 family membrane protein	3.95
HHL03_05110	*ygaZ*	Inner membrane protein YgaZ	3.67
HHL03_01725	*pstA*	high-affinity phosphate transport protein (ABC superfamily, membrane)	3.07
HHL03_15865	*oprC*	putative outer membrane copper receptor	8.02

HHL03_03645	*gntT*	Entner-Doudoroff pathway;gntT; high-affinity gluconate permease (GntP family)	2.89
HHL03_09895		MFS transporter ABC transporter permease	2.41
HHL03_02410		antibiotic transport system permease	4.84
HHL03_02415	*ybhR*	antibiotic transport system permease	5.38
HHL03_07315	*adeJ*	acridine efflux pump (RND family), partial	2.63
HHL03_07315	*adeJ*	acridine efflux pump (RND family), partial	3.10
HHL03_16150		RND type efflux pump	5.48
HHL03_16150		RND type efflux pump domain protein	3.36
HHL03_11285	*mlaE*	toluene tolerance efflux transporter (ABC superfamily, membrane)	3.56
HHL03_11290	*ttg2A*	toluene tolerance efflux transporter (ABC superfamily, ATP-bind)	3.51
HHL03_02895	*phaE*	pH adaptation potassium efflux system E transmembrane protein	2.33
HHL03_02150	*cysT*	sulfate transport protein (ABC superfamily, membrane)	74.13
HHL03_18030		thioredoxin reductase	22.93
HHL03_06620	*cysD*	sulfate adenylyltransferase subunit 2	8.62
HHL03_02140	*cysP*	sulfate transport protein (ABC superfamily, peri_bind)	5.03

Note: Positive numbers in the Fold Change column represent induction by C_{20}, negative numbers represent repression by C_{20}.

Table S3. The salt tolerance related genes in *A. pittii* sw-1.

**Locus_tag**	**Gene name**	**Gene product**
HHL03_07095	*nahP*	sodium:proton antiporter
HHL03_00240	*kdpE*	response regulator transcription factor
HHL03_00245	*kdpD*	sensor histidine kinase
HHL03_00250	*kdpC*	potassium-transporting ATPase subunit
HHL03_00255	*kdpB*	Potassium-transporting ATPase ATP-binding subunit
HHL03_00260	*kdpA*	Potassium-transporting ATPase potassium-binding subunit
HHL03_03660	*proA*	glutamate-5-semialdehyde dehydrogenase
HHL03_01970	*proB*	glutamate 5-kinase
HHL03_06955	*betT2*	choline transporter
HHL03_06960	*betT1*	BCCT family transporter
HHL03_06965	*betI*	transcriptional regulator
HHL03_06970	*betB2*	betaine-aldehyde dehydrogenase

HHL03_06975	*betA*	choline dehydrogenase
HHL03_06825	*betB1*	aldehyde dehydrogenase
HHL03_06810	*prop1*	MHS family MFS transporter
HHL03_13270	*prop2*	MFS transporter
HHL03_14275	*proP3*	MHS family MFS transporter
HHL03_09885	*otsB*	trehalose-phosphatase
HHL03_09890	*otsA*	trehalose-6-phosphate synthase

Table S4. The cold tolerance related genes in *A. pittii* sw-1.

**Locus_tag**	**Gene name**	**Gene product**
HHL03_06100	*dnaJ*	molecular chaperone
HHL03_16325	*dnaK*	molecular chaperone
HHL03_16330	*grpE*	nucleotide exchange factor
HHL03_19015	*groES*	co-chaperone
HHL03_19020	*groEL*	chaperonin
HHL03_13075	*clpB*	ATP-dependent chaperone
HHL03_03750	*secB*	protein-export chaperone
HHL03_06775	*csdA*	DEAD/DEAH box helicase family protein
HHL03_00750	*cspG1*	cold-shock protein
HHL03_00880	*cspG2*	cold-shock protein
HHL03_11665	*cspV*	cold-shock protein
